# The thermoacidophilic methanotroph *Methylacidiphilum fumariolicum* SolV oxidizes subatmospheric H_2_ with a high-affinity, membrane-associated [NiFe] hydrogenase

**DOI:** 10.1038/s41396-020-0609-3

**Published:** 2020-02-10

**Authors:** Rob A. Schmitz, Arjan Pol, Sepehr S. Mohammadi, Carmen Hogendoorn, Antonie H. van Gelder, Mike S. M. Jetten, Lena J. Daumann, Huub J. M. Op den Camp

**Affiliations:** 1 Department of Microbiology, Radboud University, Heyendaalseweg 135, NL-6525 AJ Nijmegen, The Netherlands; 2 Laboratory of Microbiology, Wageningen University and Research, Stippeneng 4, 6708 WE Wageningen, The Netherlands; 3 Department Chemie, Ludwig-Maximilians-Universität München, Butenandtstraβe 5-13, D-81377 München, Germany

**Keywords:** Microbiology, Environmental microbiology, Microbiology, Environmental microbiology

## Abstract

The trace amounts (0.53 ppmv) of atmospheric hydrogen gas (H_2_) can be utilized by microorganisms to persist during dormancy. This process is catalyzed by certain Actinobacteria, Acidobacteria, and Chloroflexi, and is estimated to convert 75 × 10^12^ g H_2_ annually, which is half of the total atmospheric H_2_. This rapid atmospheric H_2_ turnover is hypothesized to be catalyzed by high-affinity [NiFe] hydrogenases. However, apparent high-affinity H_2_ oxidation has only been shown in whole cells, rather than for the purified enzyme. Here, we show that the membrane-associated hydrogenase from the thermoacidophilic methanotroph *Methylacidiphilum fumariolicum* SolV possesses a high apparent affinity (*K*_m(app)_ = 140 nM) for H_2_ and that methanotrophs can oxidize subatmospheric H_2_. Our findings add to the evidence that the group 1h [NiFe] hydrogenase is accountable for atmospheric H_2_ oxidation and that it therefore could be a strong controlling factor in the global H_2_ cycle. We show that the isolated enzyme possesses a lower affinity (*K*_m_ = 300 nM) for H_2_ than the membrane-associated enzyme. Hence, the membrane association seems essential for a high affinity for H_2_. The enzyme is extremely thermostable and remains folded up to 95 °C. Strain SolV is the only known organism in which the group 1h [NiFe] hydrogenase is responsible for rapid growth on H_2_ as sole energy source as well as oxidation of subatmospheric H_2_. The ability to conserve energy from H_2_ could increase fitness of verrucomicrobial methanotrophs in geothermal ecosystems with varying CH_4_ fluxes. We propose that H_2_ oxidation can enhance growth of methanotrophs in aerated methane-driven ecosystems. Group 1h [NiFe] hydrogenases could therefore contribute to mitigation of global warming, since CH_4_ is an important and extremely potent greenhouse gas.

## Introduction

Hydrogen gas (H_2_) is thought to be the first available energy source on early Earth and is still ubiquitously used by a plethora of microorganisms [1, 2]. Hydrogenases convert H_2_ into two protons or vice versa and are classified based on the metals within the interior of the active site: either [NiFe], [FeFe], or [Fe] [3]. Low-affinity H_2_ oxidation is catalyzed by microbes in habitats with relatively high H_2_ availability, e.g., animal guts and leguminous soils [4, 5]. On the contrary, high-affinity microorganisms can oxidize atmospheric H_2_ (0.53 ppmv H_2_) [6]. Soils constitute the largest sink, consuming ~75% of the total atmospheric H_2_, resulting in an atmospheric turnover rate of 75 Tg H_2_ per year [6–8]. Several Actinobacteria isolated from soils are able to oxidize atmospheric H_2_ [9–11]. They have an apparent high affinity (*K*_m(app)_ ~ 10–400 nM H_2_) for H_2_. All these high-affinity actinobacterial isolates encode for the novel group 1h [NiFe] hydrogenase (Hyd-1h). This genetic finding led to the hypothesis that atmospheric H_2_ is oxidized by this type of hydrogenase in particular [10, 11]. However, microorganisms encoding the group 2a or group 1f [NiFe] hydrogenase were also shown to mediate atmospheric H_2_ oxidation [12, 13]. Nevertheless, currently it is unknown whether these hydrogenases possess an intrinsic high affinity for H_2_, or whether other cellular factors such as the respiratory chain modulate their affinity [12]. The betaproteobacterium *Ralstonia eutropha* H16 encodes for a group 1h [NiFe] hydrogenase, but this strain is unable to oxidize atmospheric H_2_ [14]. Indeed, purification of this putative high-affinity hydrogenase revealed a relatively high *K*_m_ value of 3.6 µM, reflecting a low affinity [14]. Other studies have only investigated atmospheric H_2_ oxidation in whole cells [9, 10, 15–20]. Hence, the question remains whether Hyd-1h from an organism capable of atmospheric H_2_ oxidation will show a high affinity for H_2_ after purification.

Although group 1h [NiFe] hydrogenases are predominantly encoded by Actinobacteria, examples in other phyla exist [12]. Atmospheric H_2_ oxidation has been observed in Actinobacteria, Acidobacteria, and Chloroflexi [12, 15, 18]. In addition, a conserved group 1h [NiFe] hydrogenase orthologue is highly expressed in the mud volcano-inhabiting methanotroph *Methylacidiphilum fumariolicum* SolV of the Verrucomicrobia phylum [21, 22]. Verrucomicrobia are dominant bacteria in (volcanic) soils and geothermal habitats [23, 24]. Verrucomicrobial methanotrophs are autotrophs that fix CO_2_ via the Calvin–Benson–Bassham cycle [25]. *M. fumariolicum* SolV can grow below pH 1 and is dependent on certain rare-earth elements (early lanthanides) for growth [26]. Remarkably, the Hyd-1h enables this bacterium to grow rapidly on H_2_ as sole energy source, which is in contrast to previously reported high-affinity isolates, in which the Hyd-1h is utilized during starvation to persist on atmospheric H_2_ [16, 27, 28]. The ability of methanotrophs to consume H_2_ as additional energy source provides a major advantage in ecosystems where CH_4_ availability fluctuates [29]. Previously, mixotrophy was proven to drive niche expansion of verrucomicrobial methanotrophs [30]. Methanotrophs mitigate greenhouse gas emissions from geothermal areas, which could be enhanced by hydrogenases. However, it is unknown whether methanotrophs or Verrucomicrobia are also able to oxidize atmospheric H_2_. Numerous studies have focused on the physiology of atmospheric H_2_ scavenging during dormancy [10, 11, 14, 27], but biochemical studies on the putative high-affinity Hyd-1h are scarce. Interestingly, the membrane association of the Hyd-1h seems to be essential for high-affinity atmospheric H_2_ oxidation [12, 14, 15, 27, 31]. Indeed, the cytoplasmic Hyd-1h from *R. eutropha* H16 is a low-affinity enzyme, whereas whole cell studies in microbes with membrane-associated Hyd-1h show the contrary [12, 18]. In this study, we show that methanotrophs can oxidize atmospheric H_2_, using the membrane-associated group 1h [NiFe] hydrogenase. Remarkably, the Hyd-1h of the verrucomicrobial methanotroph studied here not only enables strain SolV to oxidize atmospheric H_2_, but also to rapidly grow on geothermally derived H_2_ [22]. However, we could not show that the isolated enzyme is able to oxidize atmospheric H_2_. The isolated enzyme possesses a lower affinity for H_2_ compared with the apparent high affinity of the enzyme in association with the membrane, indicating that the membrane association is pivotal to the ability to oxidize atmospheric H_2_. We propose that the Hyd-1h provides *M. fumariolicum* SolV a survival benefit that could therefore enhance mitigation of CH_4_ emissions from methanotrophic ecosystems.

## Materials and methods

### Strain and growth conditions

*Methylacidiphilum fumariolicum* SolV cells were grown as pure culture in a 7 L bioreactor controlled by in-Control (Applikon, Delft, The Netherlands) with a working volume of 5 L. The growth medium was composed of 0.2 mM MgCl_2_·H_2_O, 0.2 mM CaCl_2_·H_2_O, 1 mM Na_2_SO_4_, 2 mM K_2_SO_4_, 2 mM (NH_4_)_2_SO_4_, and 50 µM NaH_2_PO_4_·H_2_O. The final trace element concentration was 1 µM NiCl_2_·6H_2_O, CoCl_2_·6H_2_O, NaMoO_4_·2H_2_O, and ZnSO_4_·7H_2_O, 5 µM MnCl_2_·4H_2_O and FeSO_4_·7H_2_O, 10 µM CuSO_4_·5H_2_O, and 20 nM CeCl_3_·6H_2_O. The temperature was 55 °C and maintained using a heat blanket. The pH was measured by a pH electrode and controlled at 3.0 by automatic addition of 1 M NaOH. The dissolved oxygen (DO) concentration was measured by an oxygen electrode. The airflow was regulated to maintain a DO concentration of 1% air saturation. The reactor was stirred at 500 rpm using a stirrer with two Rushton impellers. The reactor was supplied with 70 mL min^−1^ CO_2_–Ar (5%:95% (v/v)), 10 mL min^−1^ CH_4_–CO_2_ (95%:5% (v/v)) and 6 mL min^−1^ H_2_.

Cells (and membranes) used to investigate atmospheric H_2_ oxidation and kinetics were gained from a culture grown on H_2_ limitation. Cells in the 0.5 L bioreactor (Applikon) were supplied with a mixture of argon, H_2_ (0.7% (v/v)), and CO_2_ (10% (v/v)) at 5 mL min^−1^. Five millimolars NaNO_3_ was used as nitrogen source instead of ammonium. The DO concentration was kept between 0.2 and 1% O_2_ saturation.

### Gas chromatography

Atmospheric H_2_ was measured on a CompactGC (Global Analyser Solutions, Breda, The Netherlands) with a pulsed discharge ionization detector with an input range of 8 nA. One hundred twenty milliliters serum bottles containing 10 mL cell suspension (OD_600_ = 0.12) were used, capped with a rubber stopper. The heat-killed control was autoclaved at 121 °C for 15 min. The bottles were made anoxic by flushing with N_2_, after which 1 mL O_2_ and 3 mL CO_2_ were added to the headspace. H_2_ was added from a stock diluted in N_2_ to obtain trace concentrations. To test whether the purified Hyd-1h could oxidize atmospheric H_2_, 120 serum bottles were filled with 5 mL 50 mM KP_i_ (pH 7.0), 500 µM nitroblue tetrazolium. 0.3 mg mL^−1^ bovine serum albumin was present in the bottles to create a stable protein environment. The bottles were made anoxic by flushing with N_2_. The assay was initiated by addition of 30 µg purified hydrogenase. Bottles containing cells or pure enzyme were incubated at 55 and 40 °C, respectively, at 300 rpm. Biological triplicates were used and for each data point 0.2 mL headspace was sampled.

### Preparation of cell fractions and enzyme purification

Cells were harvested by centrifugation (15 min, 5000 × *g*, 4 °C), resuspended in 20 mM bis-Tris buffer (pH 7.0), and passed through a French pressure cell (120 MPa, three times; American Instrument Company, Silver Spring, MD, USA). Hereafter, the crude extract (CE) was obtained by collecting the supernatant after centrifugation (10 min, 10,000 × *g*, 4 °C). Subsequently, the CE was centrifuged (1 h, 137,000 × *g*, 4 °C). The pellet, containing the membrane proteins and membranes (membrane fraction; MF), was homogenized using a 15 mL Potter-Elvehjem tissue grinder (DWK Life Sciences, Mainz, Germany) and subsequently mixed with 1% (v/v) n-dodecyl-β-maltoside for 1 h at room temperature (RT) by gentle stirring. This suspension was centrifuged again (1 h, 137,000 × *g*, 4 °C). The supernatant containing the solubilized MF (SMF) was used for protein purification, carried out aerobically at RT on an Äktapurifier (GE Healthcare Bio-Science AB, Uppsala, Sweden). All buffers contained 0.02% (v/v) n-dodecyl-β-maltoside. Hyd-1h was purified using three different consecutive columns. First, the SMF was loaded onto a prepacked Q Sepharose column (GE Healthcare, Chicago, IL, USA), equilibrated with 20 mM bis-Tris (pH 7.0). Then, the sample was loaded and the column was washed with 20 mM bis-Tris 100 mM NaCl (pH 7.0). Hereafter, the column was washed with 20 mM bis-Tris 200 mM NaCl (pH 7.0) and the most active fractions were pooled. Subsequently, the buffer of the sample was exchanged to 20 mM KP_i_ (pH 7.0) and the concentrated sample was loaded onto a prepacked CHT ceramic hydroxyapatite column (Bio-Rad, Hercules, CA, USA), equilibrated with 20 mM KP_i_ (pH 7.0). Hereafter, a gradient of 30 column volumes to 500 mM KP_i_ (pH 7.0) was performed. The most active fractions were pooled and the buffer of the sample was exchanged back to 20 mM bis-Tris (pH 7.0). Finally, the sample was loaded onto a prepacked TSKgel DEAE-5PW column (Merck, Darmstadt, Germany), equilibrated with 20 mM bis-Tris (pH 7.0). The hydrogenase was eluted during a gradient of 20 column volumes to 20 mM bis-Tris 300 mM NaCl (pH 7.0). The purified enzyme (in 20 mM bis-Tris buffer, 200 mM NaCl, 0.02% (v/v) n-dodecyl-β-maltoside, pH 7.0) was snap-frozen in liquid nitrogen and stored at −80 °C. Protein concentrations of the different samples were measured using the BCA method (Thermo Fisher Scientific, Waltham, MA, USA) with bovine serum albumin as standards.

### Kinetic studies using membrane-inlet mass spectrometry (MIMS)

Liquid concentrations of H_2_ were measured by MIMS (HPR40, positive ion counting detector, Hiden Analytical, Warrington, UK) in a 10 mL chamber (Fig. S3). The MIMS probe was inserted inside of the bottom part just above the glass stirrer bar (1000 rpm). The probe was mounted with a 10 µm thin silicon (Hiden Analytical) or PTFE (Hansatech, Pentney, UK) membrane (8 mm^2^). The liquid was equilibrated with the desired gas by bubbling the gas into the liquid via a metal capillary inserted via the central hole in the piston. When equilibrated, the capillary was removed and the piston was lowered until the liquid filled up the central hole (0.8 mm diameter) and ensuring no gas bubbles were trapped inside the chamber. The liquid volume was between 8 and 9 mL. All additions were done via the same hole by gastight syringes (Hamilton, Reno, NV, USA). Signals of mass 2 were not only from H_2_ but resulted also from water and gases such as CO_2_. Therefore, the vacuum line (1/8 in. stainless steel tubing) connecting the membrane probe with the mass spectrometer had a coiled part (three windings of 8 cm diameter) inserted in liquid nitrogen to trap water vapor and other gases that pass the silicon membrane. In this way, the background mass 2 signal was minimized. For maximum sensitivity, the electron emission current was optimized at 400 µA. For calibrations, known amounts of gas-saturated water were administered from 50 mL serum bottles containing 10 mL water. The headspaces were exchanged and pressurized with pure gas at 1–1.3 bar and stoppered by butyl rubber stoppers. For water, a solubility of 807 µM at RT was used [32]. For O_2_, a solubility of 1.35 mM at atmospheric pressure was used. In the experiment with whole cells, the medium used was identical to the growth medium and the pH value was as during growth. In the experiment with membranes, 20 mM bis-Tris buffer (pH 7.0) was used. The initial DO concentration was set at 1% by bubbling with a mixture of argon, CO_2_ (5%), and O_2_ (1%). During respiration of H_2_, the O_2_ concentration was maintained between 0.5 and 1% DO by adding O_2_-saturated medium. Either cells from the chemostat or the MF were used at 50 and 40 °C, respectively. With the MF, either 110 µM methylene blue (MB) or 100 µM Wurster’s blue (WB) was used as electron acceptor. When working with the isolated enzyme, 100 µM nitroblue tetrazolium was used as electron acceptor. The cell was thoroughly flushed with argon and 0.3 mg mL^−1^ bovine serum albumin was supplemented to stabilize the 250 ng isolated enzyme used in the experiment. After the data were gathered, the curve was fitted according Michaelis–Menten kinetics. From this fit, the maximum oxidation rates (*V*_max_) and (apparent) affinity constants (*K*_m_) could be determined.

### Gel electrophoresis and mass spectrometry

SDS-polyacrylamide gels for molecular mass determination of the proteins were made and run as described by [33]. Five microliters PageRuler Plus Prestained Protein Ladder (Thermo Fisher Scientific) was used as molecular ladder. To identify bands on the SDS-polyacrylamide gels, samples were analyzed with the use of matrix-assisted laser desorption/ionization time-of-flight mass spectrometry. Therefore, samples were prepared and analyzed as described by [33]. A calibrant protein mixture was prepared from 10 pmol stocks by mixing 1 µL adrenocorticotropic hormone, 1.5 µL synthetic peptide P14R, 2 µL bradykinin, 2 µL angiotensin II, and 6.5 µL 4-hydroxy-α-cyanocinnamic acid matrix. One microliter of this mixture was used for analysis. Gene identifiers in the text refer to the Genoscope Platform. The GenBank identifiers for the large and small subunit of Hyd-1h are MFUMSOLV_RS08465 and MFUMSOLV_RS08470, respectively.

### Spectrophotometric enzyme assays

Activity assays were performed in 1.4 mL 10.00 mm quartz cuvettes at 50 °C and initiated by addition of the protein sample or cell fraction. The cuvettes were filled with H_2_-saturated 20 mM bis-Tris (pH 7.0) and 1 mM nitroblue tetrazolium and immediately closed with a plastic cap at RT. A small headspace with ambient air was always present. Cuvettes containing buffer were preincubated for 5 min at the right temperature at which the experiment would take place, while the background signal was measured. Reduction of nitroblue tetrazolium was followed at 593 nm. The thermostability assay was performed at 50 °C with purified enzyme preincubated at 70, 80, and 85 °C for 30 min. The temperature optimum experiment was measured from 30 to 100 °C with increments of 10 °C. The pH optimum experiment was measured from pH 4 to 10 in a Britton–Robinson pH system at 50 °C containing 40 mM phosphoric acid, boric acid, and acetic acid [34]. In spectrophotometry assays, initial rates in the first 30 s were used to determine the H_2_ oxidation rates.

### Circular dichroism

The J-810 circular dichroism spectrometer (Jasco, Oklahoma City, USA) was used to observe changes in secondary structure upon temperature change. A scanning speed of 100 nm min^−1^, a data pitch of 0.1 nm, a data integration time of 1 s, and a bandwidth of 1.0 nm were used. Baseline spectra of the buffer at different temperatures were recorded and subtracted from the protein spectrum at the corresponding temperature. Spectra were obtained from 200 to 260 nm, measured after 1, 30, and 60 min incubation at 95 °C. The buffer of the sample was exchanged to 20 mM phosphate buffer (pH 7.0) to reduce background signal.

## Results

### *M. fumariolicum* SolV oxidizes subatmospheric H_2_ with apparent high affinity

**Fig. 1:**
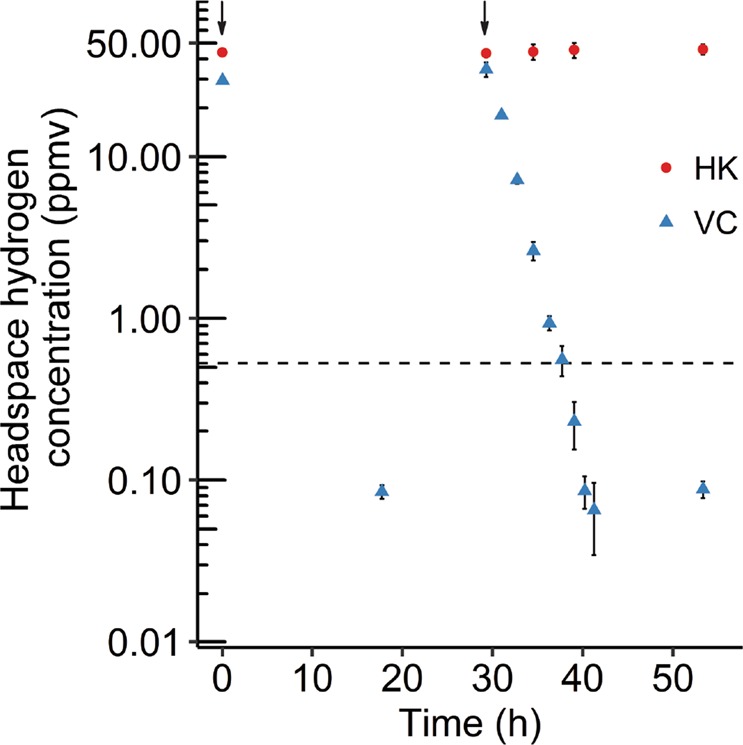
Subatmospheric H_2_ oxidation by Hyd-1h in cells of *Methylacidiphilum fumariolicum* SolV. The dashed line indicates the atmospheric H_2_ concentration (0.53 ppmv). The arrows indicate when hydrogen gas was added. One hundred twenty milliliters capped serum bottles were inoculated with 10 mL (OD_600_ = 0.12) heat-killed cells (HK) or viable cells (VC). At 29 h, H_2_ was again supplemented to VC. Error bars indicate standard deviations (*n* = 3).

*M. fumariolicum* SolV cells incubated at 55 °C oxidized 29 ± 1.3 ppmv H_2_ to well below the atmospheric H_2_ threshold within 18 h (Fig. 1). A second addition of H_2_ after 29 h again resulted in the rapid first-order oxidation of H_2_ (3.15 ± 0.32 ppmv H_2_ h^−1^) below the atmospheric H_2_ threshold. Heat-killed cells did not show any H_2_ uptake, confirming the process to be biotic. Kinetic parameters of H_2_ consumption were determined in a closed cell with a diluted culture and no gas phase by measuring H_2_ with MIMS. This technique was conducted to avoid mass transfer problems and the limitation in sampling encountered with headspace measurements for gas chromatography. The maximum H_2_ oxidation rate (*V*_max(app)_) was measured to be 340 nmol H_2_ min^−1^ mg DW^−1^ and the cells have an apparent high affinity (*K*_m(app)_) for H_2_ of 195 ± 10 nM (*n* = 8; Fig. S1). To ensure that only Hyd-1h was responsible for the observed H_2_ consumption down to the threshold level of the MIMS (20 nM), the cells used were cultivated in a chemostat under such conditions that only Hyd-1h (MfumV2_0978-9) was expressed, but not the oxygen-sensitive group 1d [NiFe] hydrogenase (MfumV2_1564-5) [22]. Similarly, experiments were performed in the presence of at least 0.2% O_2_, so that traces of the group 1d [NiFe] hydrogenase would be inactive [22]. The apparent kinetic parameters strongly suggest that Hyd-1h is solely accountable for subatmospheric H_2_ oxidation in the methanotroph *M*. *fumariolicum* SolV.

### Membrane-associated Hyd-1h possesses an apparent high affinity for H_2_

**Fig. 2:**
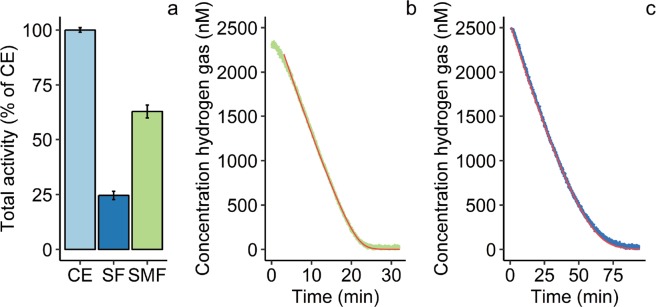
H_2_ oxidation by Hyd-1h. **a** Total H_2_-oxidizing activity of the soluble fraction (SF) and the solubilized membrane fraction (SMF) in % of the total activity of the crude extract (CE). Activity was measured spectrophotometrically at 593 nm at 50 °C as reduction of the electron acceptor nitroblue tetrazolium. Error bars indicate standard deviations (*n* = 3). **b** High-affinity H_2_ oxidation by the membrane-associated Hyd-1h with methylene blue as electron acceptor. **c** H_2_ oxidation by the purified Hyd-1h with nitroblue tetrazolium as electron acceptor. Hydrogen gas was measured at 40 °C by the membrane-inlet mass spectrometer (MIMS). To determine kinetic parameters, data points were fitted according to Michaelis–Menten kinetics. The red lines show the fitted curves.

Analysis of the two genes (MfumV2_0978-9) encoding Hyd-1h revealed no transmembrane helices nor a signal peptide, suggesting that the enzyme resides within the cytoplasm without membrane anchor [35, 36]. Nevertheless, the total activity measured in the SMF exceeds that of the soluble fraction (SF) more than 2.5-fold (Fig. 2a). This membrane association could enable energy conservation and could be formed through a currently unknown physiological redox partner protein [37]. The activity of the SF could be a combination of Hyd-1h and the putative NADH-reducing group 3b [NiFe] hydrogenase (MfumV2_2420-1; [30]). However, expression of Hyd-1h is 20–40-fold higher compared with the expression of the group 3b [NiFe] hydrogenase at various conditions [22]. In addition, strain SolV was grown at such an O_2_ concentration that the group 1d [NiFe] hydrogenase is barely expressed [22]. Therefore, the activity of the CE can be primarily attributed to Hyd-1h. The enzyme is bound weakly to the membrane so that the activity is found in both SF and SMF.

The group 1h [NiFe] hydrogenase mediates atmospheric H_2_ oxidation, but it is still unknown whether the isolated enzyme has a high affinity for H_2_ or that this affinity is modulated by other cellular factors [12, 18, 22]. Since the enzyme tends to associate with the membrane, which seems to be important for enzyme functioning, kinetics would be most accurately determined by mimicking these conditions [14]. To determine kinetic parameters of the membrane-associated hydrogenase only, the enzyme needed to be decoupled from the remainder of the electron transport chain. For this purpose, membranes of strain SolV were isolated and MB (redox potential = +10 mV) was used as electron acceptor [27]. As a consequence, electrons released by Hyd-1h would be taken up before flowing to electron transport chain proteins with a higher redox potential (e.g., complex III and the terminal oxidase). When enzyme kinetics were assayed with MIMS, we could show that the membrane-associated Hyd-1h possesses a high apparent affinity (*K*_m(app)_) of 140 ± 10 nM (*n* = 4) and a maximum velocity (*V*_max(app)_) of 38 nmol H_2_ min^−1^ mg protein^−1^ (Fig. 2b). Furthermore, WB (redox potential = + 276 mV) was used as electron acceptor. When WB was used, a significantly higher affinity of 90 ± 5 nM (*n* = 4) was determined. However, the maximum velocity decreased severely after subsequent H_2_ additions after H_2_ depletion (from 67 to 37 nmol H_2_ min^−1^ mg protein^−1^)_._ On the contrary, maximum rates did not decrease over time when MB was used as electron acceptor. Apparently, different artificial electron acceptors reveal different kinetic parameters. The parameters of the membrane-associated Hyd-1h were most stably determined with MB as electron acceptor.

### Hyd-1h purified from native biomass is highly thermostable

To investigate enzyme characteristics such as thermostability, the enzyme needs to be devoid of other proteins that could interfere. Therefore, the enzyme was purified 45-fold from the SMF to a specific activity of 18.4 µmol H_2_ min^−1^ mg protein^−1^ (Table S1). The specific activity of the SMF is more than twice as high as the specific activity of the CE. Hence, Hyd-1h was purified approximately 100-fold from the CE. Two bands with apparent sizes of 65 and 40 kDa could be observed on a denaturing SDS-polyacrylamide gel after purification (Fig. S2). The large and small band were identified with matrix-assisted laser desorption/ionization mass spectrometry as the large (HhyL; MfumV2_0979) and small (HhyS; MfumV2_0978) subunit of Hyd-1h, respectively. Whereas cells of strain SolV are able to oxidize 30 ppmv H_2_ to well below the atmospheric H_2_ threshold, the purified enzyme was unable to do so. The activity of the isolated enzyme diminishes over time, which could be largely overcome by using a concentration of 0.3 mg mL^−1^ bovine serum albumin to create a stabilizing protein environment. The affinity for H_2_ of the isolated enzyme was determined in the MIMS cell with nitroblue tetrazolium as electron acceptor. Prior to the actual kinetic measurements, 3 µM H_2_ was oxidized to approximately 500 nM H_2_ over time and replenished to 3 µM H_2_ several times, because we observed the enzyme needed to be activated to show a stable oxidation rate. Hereafter, the oxidation of 2.7 µM H_2_ was followed over time (Fig. 2c). From this, a *K*_m_ value of 300 nM could be determined, which is higher than the values determined for whole cells and the membrane-associated Hyd-1h. We observed that the enzyme was stable over periods of several hours when H_2_ was present at saturating concentrations. However, when H_2_ had become depleted and the MIMS cell was again supplemented with 3 µM H_2_, a significantly lower activity and lower affinity were observed. In the MIMS cell, submicromolar levels of O_2_ are always present due to leakage. Only in the presence of the reducing agent dithiothreitol the oxygen problem could be overcome temporarily. Still, the activity of the isolated enzyme diminished over time in a buffered assay when H_2_ had become depleted. In conclusion, the isolated enzyme showed a lower affinity for H_2_ compared with the membrane-associated enzyme, which could be explained by oxygen poisoning upon H_2_ depletion, by the disconnection from the membrane or by a combination of both.

**Fig. 3:**
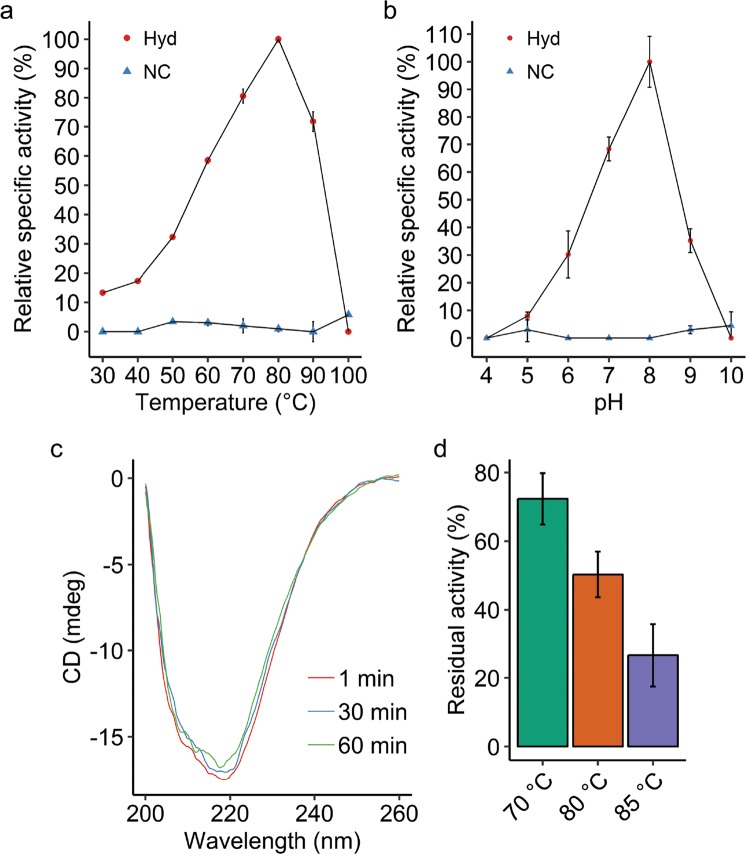
Properties of the purified Hyd-1h at a range of conditions. **a**, **b** Relative specific activity (in % of the optimum activity) of the isolated hydrogenase (Hyd) and the control without enzyme (NC) within a temperature range of 30 to 100 °C (**a**) and within a pH range of 4 to 10 at 50 °C (**b**). **c** Circular dichroism spectrum from 200 to 260 nm measured after 1, 30, and 60 min incubation at 95 °C in 20 mM phosphate buffer (pH 7.0). **d** Residual activity (% of activity prior to heating) of the enzyme at 50 °C after incubation for 30 min at 70, 80, or 85 °C. Error bars indicate standard deviations (*n* = 3).

In order to determine the temperature optimum of the purified enzyme, the activity was measured spectrophotometrically in a range from 30 to 100 °C (Fig. 3a). A clear linear increase with every increment of 10 °C was observed from 40 to 80 °C. At the temperature optimum of 80 °C, the specific activity is approximately 7.5-fold higher compared with the specific activity at 30 °C. At 90 °C, roughly 30% of activity is lost in comparison to the optimum temperature, whereas at 100 °C all activity is lost. To determine the optimum pH of the hydrogenase, the activity was tested within a pH range of 4 to 10 (Fig. 3b). The acidity determines the charge of the amino acid residues and therefore has a profound effect on its folding and therefore its activity. A pH of 4 is too acidic for the enzyme to function, whereas a pH of 10 is too alkaline. A relative specific activity of 68 and 35% at respectively pH 7 and pH 9 was measured compared with the activity at the optimum pH of 8. To test the thermostability of the enzyme, the secondary structure and the activity were assessed at high temperatures. Remarkably, enzyme folding is recalcitrant to extremely high temperature, since the majority of the enzyme remains folded after at least 1 h at 95 °C as shown by circular dichroism measurements (Fig. 3c). Accordingly, the enzyme is able to largely retain its activity for a prolonged period at high temperature. Even at 80 °C the purified Hyd-1h from *M. fumariolicum* SolV retains a half-life stability of 30 min (Fig. 3d).

## Discussion

In this study we show that the membrane-associated group 1h [NiFe] hydrogenase possesses a high apparent affinity for H_2_, which enables the thermoacidophilic methanotroph *M. fumariolicum* SolV to oxidize subatmospheric H_2_. Previous studies on atmospheric H_2_ oxidation by soil bacteria had already revealed apparent kinetic parameters (*K*_m(app)_ ~ 10–400 nM H_2_) associated with this process [10, 12]. However, since these parameters were obtained in experiments with whole cells, it was unclear whether these low apparent affinity constants are attributable to intrinsic properties of the enzyme or to other cellular components, such as electron transport chain proteins [12]. We have shown that the membrane-associated Hyd-1h has a high apparent affinity (*K*_m(app)_ = 140 ± 10 nM) for H_2_, but that this affinity is lowered when the isolated enzyme is disconnected from the membrane (*K*_m_ = 300 nM). We therefore suggest that the membrane association of the group 1h [NiFe] hydrogenase is pivotal for enzyme functioning and hence for atmospheric H_2_ oxidation.

*M. fumariolicum* SolV is able to grow on CH_4_ and H_2_ concurrently as well as alternately [21, 22]. Moreover, growth on H_2_ is possible by solely utilizing Hyd-1h, which is in contrast to other microorganisms harboring this enzyme, where it aids in cometabolism of H_2_ for survival, not growth [15, 18, 22]. In *Mycobacterium smegmatis* mc^2^155, cells expressing Hyd-1h consume H_2_ with a maximum velocity of 2.5 pmol H_2_ min^−1^ mg DW^−1^, which is much slower than the 340 nmol H_2_ min^−1^ mg DW^−1^ determined in this study [12]. Therefore, Hyd-1h of strain SolV seems to be tuned toward rapid oxidation and growth on H_2_, whereas Actinobacteria and Chloroflexi use this enzyme to scavenge low concentrations of H_2_ to persist during dormancy [12, 15, 18]. However, apparent kinetic parameters of strain SolV cells are similar to those of other microorganisms oxidizing atmospheric H_2_. It was hypothesized that certain features in the respiratory chain or redox metabolism of *M. smegmatis* could contribute to the high affinity for H_2_ [12]. Indeed, the affinity for H_2_ is lowered when the enzyme is isolated and thus disconnected from the electron transport chain. Still, it is conceivable that we have not yet found the optimal in vitro conditions to reveal the true characteristics of the isolated enzyme. The apparent affinity of 190 ± 10 nM in cells of strain SolV significantly differs from the 0.6 µM measured by gas chromatography before, which could be attributed by limitations in gas–liquid mass transfer in the former study [22]. In general, H_2_ is a key driver for microbial activity in geothermal areas and emitted at higher concentrations than the atmospheric H_2_ concentration, although emissions can greatly vary spatiotemporally [6, 30, 38–40]. It is therefore conceivable that extremophilic methanotrophs such as strain SolV utilize Hyd-1h to rapidly oxidize H_2_ as alternative energy source as well as persist on atmospheric H_2_ in the natural environment.

This study describes the first purification of a putative high-affinity hydrogenase from an organism able to oxidize atmospheric H_2_. Remarkably, Hyd-1h from *M. fumariolicum* SolV possesses distinct properties in comparison with the orthologue from *Ralstonia eutropha* H16, which was obtained by purification after homologous expression [14]. Because of its low-affinity Hyd-1h (*K*_m_ = 3.6 µM), this betaproteobacterium is unable to oxidize atmospheric H_2_ [14]. Hyd-1h of *M. fumariolicum* SolV does not possess a membrane anchor but the enzyme is clearly associated with the membrane. This is in agreement with other bacteria harboring Hyd-1h, e.g., *Mycobacterium smegmatis* and *Pyrinomonas methylaliphatogenes* [12, 18]. This membrane association of Hyd-1h could be a determining factor in the high affinity for H_2_, as in *R. eutropha* H16 the low-affinity enzyme was purified from the cytoplasm [14, 27]. Greening et al. have suggested a [2Fe2S] protein encoded in the Hyd-1h operon serves as intermediate redox partner. Indeed, these proteins are important in *Mycobacterium smegmatis*, but they are not conserved in all microorganisms that contain Hyd-1 and can oxidize atmospheric H_2_ [41]. In addition, in the acidobacterium *Pyrinomonas methylaliphatogenes* the electron flow could be facilitated by hypothetical proteins in the Hyd-1h operon [18]. However, these homologous genes are not encoded in the genome of *M. fumariolicum* SolV. Moreover, in its Hyd-1h operon two hypothetical membrane proteins are encoded (MfumV2_0985-6) that are absent in Actinobacteria and Acidobacteria, but lack iron–sulfur clusters. The apparent affinity of the membrane-associated Hyd-1h (*K*_m(app)_ = 140 nM) is comparable to apparent affinities in cells of atmospheric H_2_-oxidizing strains [10, 18, 27]. The isolated Hyd-1h in vitro, however, does not show a comparable high affinity for H_2_ when disconnected from the membrane and is unable to oxidize subatmospheric H_2_.

Purification of the enzyme allowed us to investigate the proposed characteristics (such as thermostability) of Hyd-1h [27]. Indeed, the enzyme is extremely thermostable, possessing a tenfold longer half-life time at 80 °C compared with the purified thermostable enzyme of *R. eutropha* H16 [14]. In addition, biophysical studies on the enzyme in UV-range revealed the enzyme to retain its folding at least for 1 h at 95 °C. The high thermostability could be explained by a combination of the proposed thermophilic origin of the enzyme and environmental pressure [27, 42]. Since *M. fumariolicum* SolV thrives in thermophilic habits instead of mesophilic habitats as *R. eutropha* H16 does, high temperature might be an environmental pressure for an even more heat-resistant enzyme. Studies on moderate temperature soils have shown cessation of atmospheric H_2_ consumption above 40 °C [43]. However, this is likely due to heat sensitivity of the organism and not of the enzyme, since atmospheric H_2_ oxidation at high temperatures was shown in Chloroflexi and Acidobacteria strains [15, 18]. One of the reasons postulated for enzyme thermostability is the tight dimer packing of the enzyme [44]. The pH range in which the purified enzyme functions is similar to the purified protein of *R. eutropha* H16 at neutral range. However, the latter showed increased activity at pH > 10, outside of the physiological range [14]. In accordance with other studies, Hyd-1h of *M. fumariolicum* SolV remains active under ambient oxygen concentrations in the cell [22]. Furthermore, it could also be purified under aerobic conditions, while retaining catalytic activity. Interestingly, the enzyme from *R. eutropha* H16 was shown to be completely insensitive to oxygen. However, this was measured in the presence of high concentrations of H_2_ that keep the enzyme in a reduced state. Group 1h [NiFe] hydrogenases are thought to possess specific mechanisms to cope with high O_2_ levels, such as a narrow gas tunnel and a specific aspartate residue coordinating the proximal [4Fe4S] cluster [44]. However, we observed that in the absence of H_2_, the isolated enzyme seems impeded by oxygen. Long-term storage was therefore done under anoxic conditions. Most hydrogenases are inhibited by the presence of O_2_, therefore studying hydrogenases that show high tolerance to oxygen are interesting to study from a biotechnological perspective [45].

Utilization of both H_2_ and CH_4_ as energy source renders *M. fumariolicum* SolV metabolically flexible in ecosystems with fluctuations in H_2_ and CH_4_ emissions [22, 29, 38, 39]. In methanotrophs, Hyd-1h could therefore mitigate the emission of greenhouse gases from geothermal systems, especially in the presence of high O_2_ concentrations. In addition, atmospheric H_2_ controls the levels of atmospheric hydroxyl radicals and in turn the formation of methane [46]. We suggest strain SolV utilizes this enzyme to rapidly grow on H_2_ and to scavenge atmospheric H_2_ during persistence. The high affinity for H_2_ is likely an ancient trait of the enzyme, used by soil-dwelling microorganisms in particular that persist on this ubiquitous atmospheric energy source [27]. We conclude that Hyd-1h has an important role in hostile methanotrophic systems for extremophilic methanotrophs to thrive and that at least a major part of atmospheric H_2_ could be oxidized due to the membrane-associated Hyd-1h. Further work is needed to assess in which ecosystems microorganisms are actively oxidizing atmospheric H_2_ and which hydrogenases are employed to catalyze this reaction.

## Supplementary information

Supplementary material
