# Supplementary material for: The thermoacidophilic methanotroph Methylacidiphilum fumariolicum SolV oxidizes subatmospheric H2 with a high-affinity, membrane-associated [NiFe] hydrogenase
Source: ISME J. 2020 Feb 10;14(5):1223–32. doi: 10.1038/s41396-020-0609-3 (PMC7174314; doi:10.1038/s41396-020-0609-3)
Supplement: Supplementary file 1 — Supplementary material [file 41396_2020_609_MOESM1_ESM.pdf]

Supplementary information

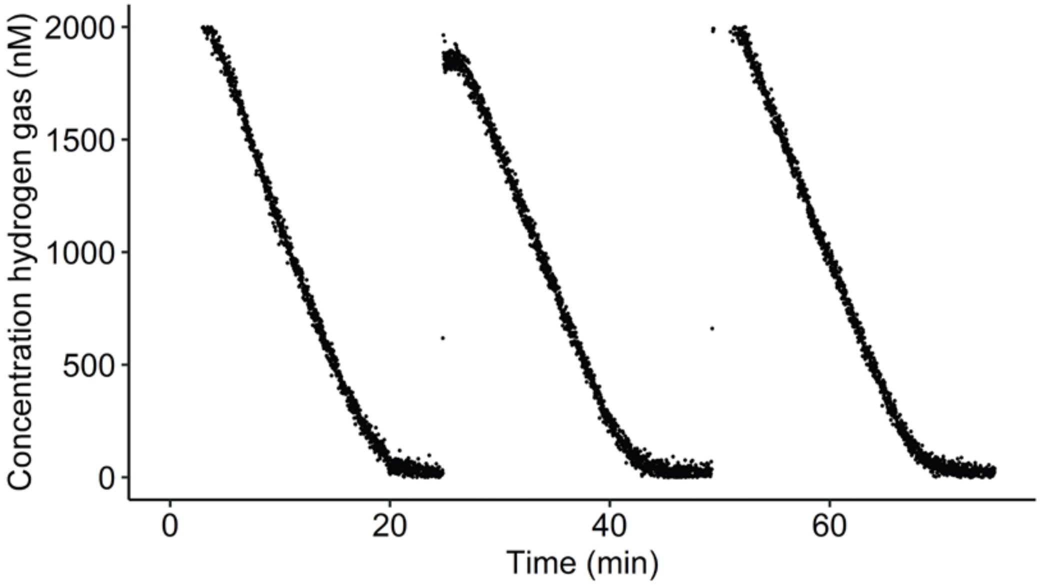

Fig. S1 H_2 oxidation by cells of *Methylacidiphilum fumariolicum* SolV measured by membrane-inlet mass spectrometry (MIMS). Cells from the H_2-limited chemostat were used (OD_{600} = 0.1; 100 µL) in the 9 mL MIMS cell at 50 °C. After 25 and 49 minutes, again H_2 was added to the MIMS cell. To determine kinetic parameters, data points were fitted according to Michaelis-Menten kinetics.

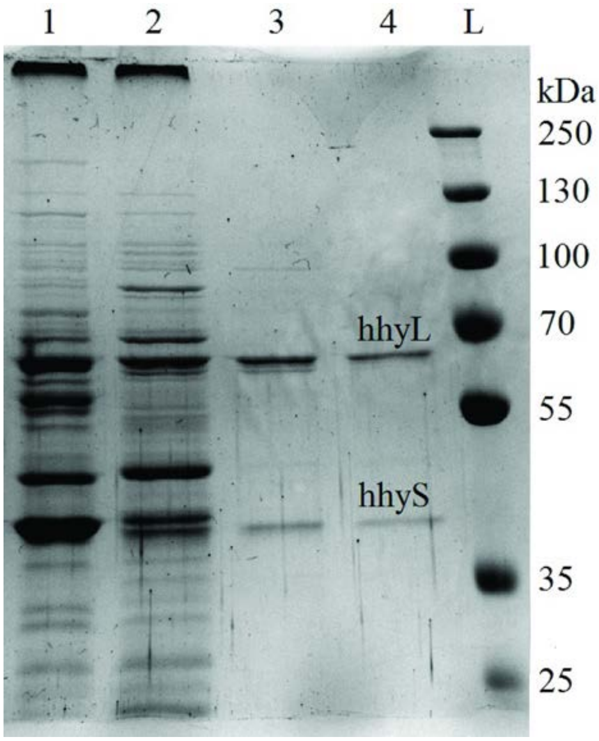

Fig. S2 SDS-polyacrylamide gel showing the purification of Hyd-1h from *Methylacidiphilum fumariolicum* SolV. Lane 1: solubilized membrane fraction containing all membrane proteins. Lane 2: pooled fractions eluted from the Q Sepharose column. Lane 3: pooled fractions eluted from the CHT Ceramic Hydroxyapatite column. Lane 4: purified Hyd-1h eluted from the TSKgel DEAE-5PW column. L: protein ladder of different masses in kDa. The large (hhyL; MfumV2_0979) and small (hhys; MfumV2_0978) subunit of Hyd-1h were detected by matrix-assisted laser desorption/ionization time-of-flight mass spectrometry.

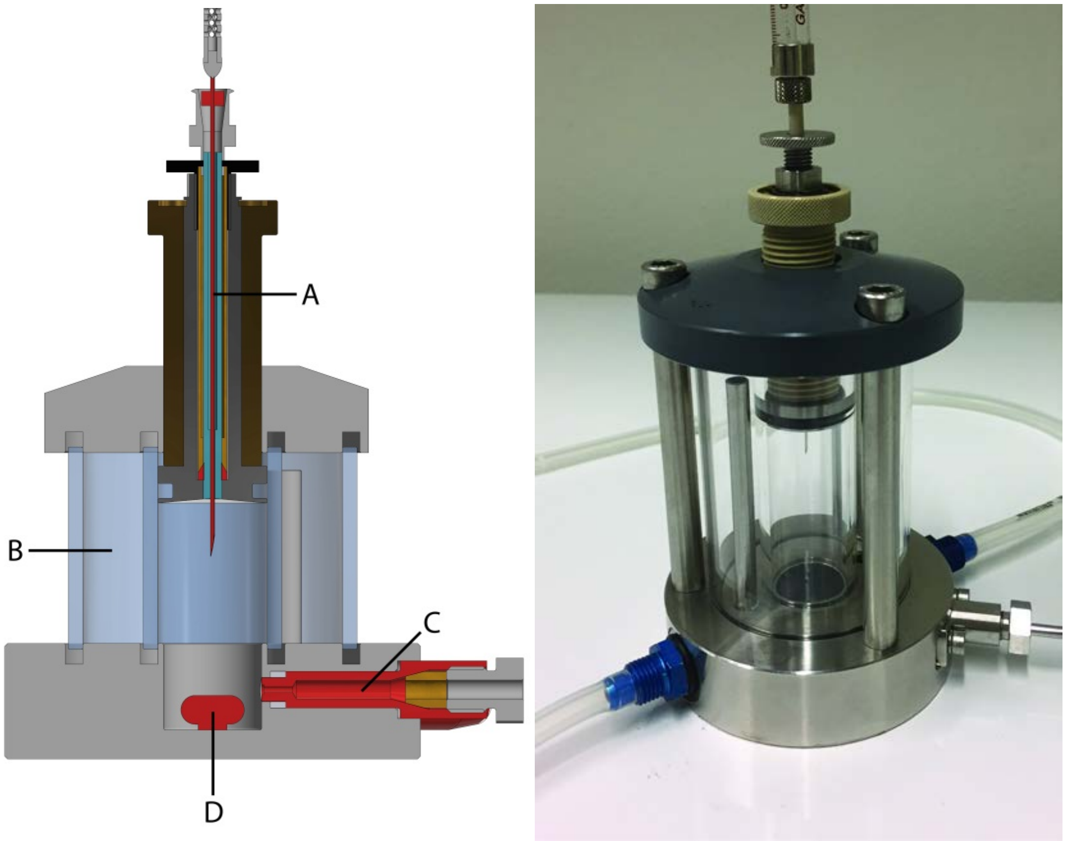

Fig. S3 Membrane-inlet mass spectrometry (MIMS) cell used for kinetics studies. Schematic figure (left) and photograph (right) of the MIMS cell. A: Piston with a needle inlet, B: water jacket for temperature regulation, C: MIMS probe connected to the cell via a membrane (8 mm^2) that allows diffusion of gases, D: magnetic stirrer bar.

Table S1 Purification table of Hyd-1h from *Methylacidiphilum fumariolicum* SolV. SMF = solubilized membrane fraction; Q = QSepharose column; HA = Hydroxyapatite column; DEAE = TSK diethylaminoethyl column. Activity was measured spectrophotometrically at 593 nm at 50 °C as reduction of the electron acceptor nitroblue tetrazolium.

**Fraction**	**Protein amount** (mg)	**Total activity** (U*)	**Specific activity** (U · mg protein^{-1})	**Purity** (-fold)	**Yield** (%)
SMF	177	73.7	0.41	1	100
Q	4.2	21.9	5.2	13	30
HA	1.0	9.4	9.3	23	13
DEAE	0.2	3.9	18.4	45†	5

* One unit (U) is defined as the oxidation of 1 µmol H_2 · min^{-1}.

† If purity is calculated from the crude extract (CE), the purification is approximately 100-fold.
